# Prevalence and factors associated with covert contraceptive use in Kenya: a cross-sectional study

**DOI:** 10.1186/s12889-021-11375-7

**Published:** 2021-07-05

**Authors:** Catherine Akoth, James Odhiambo Oguta, Samwel Maina Gatimu

**Affiliations:** 1 Institute of Tropical and Infectious Diseases, University of Nairobi, Nairobi, Kenya; 2 School of Public Health, University of Nairobi, Nairobi, Kenya; 3 School of Economics, University of Nairobi, Nairobi, Kenya

**Keywords:** Kenya, Covert contraceptive use, Family planning, Autonomy, Secret use, Male involvement, injectaAles, PMA survey, Decision making

## Abstract

**Background:**

Family planning (FP) is a key intervention for preventing unplanned pregnancies, unsafe abortions, and maternal death. Involvement of both women and their partners promotes contraceptive acceptance, uptake and continuation, couple communication and gender-equitable attitude. Partner involvement is a key strategy for addressing about 17.5% of the unmet needs in FP in Kenya. This study assessed the prevalence and factors associated with covert contraceptive use (CCU) in Kenya.

**Methods:**

We used data from the sixth and seventh rounds of the performance monitoring for accountability surveys. We defined CCU as “the use of contraceptives without a partner’s knowledge”. We used frequencies and percentages to describe the sample characteristics and the prevalence of CCU and assessed the associated factors using bivariate and multivariable logistic regressions.

**Results:**

The prevalence of CCU was 12.2% (95% CI: 10.4–14.2%); highest among uneducated (22.3%) poorest (18.2%) and 35–49 years-old (12.8%) women. Injectables (53.3%) and implants (34.6%) were the commonest methods among women who practice CCU. In the bivariate analysis, Siaya county, rural residence, education, wealth, and age at sexual debut were associated with CCU. On adjusting for covariates, the odds of CCU were increased among uneducated women (aOR 3.79, 95% CI 1.73–8.31), women with primary education (aOR 1.86, 95% CI 1.06–3.29) and those from the poorest (aOR 2.67, 95% CI 1.61–4.45), poorer (aOR 1.79, 95% CI 1.05–3.04), and middle (aOR 2.40, 95% CI 1.52–3.78) household wealth quintiles and were reduced among those with 2–3 (aOR 0.49, 95% CI 0.33–0.72) and ≥ 4 children (aOR 0.62, 95% CI 0.40–0.96). Age at sexual debut (aOR 0.94, 95% CI 0.89–0.99) reduced the odds of CCU.

**Conclusion:**

About one in 10 married women in Kenya use contraceptives covertly, with injectables and implants being the preferred methods. Our study highlights a gap in partner involvement in FP and calls for efforts to strengthen their involvement to increase contraceptive use in Kenya while acknowledging women’s right to make independent choices.

## Introduction

Kenya has a high fertility rate of 3.9 births per woman [1], a teenage pregnancy rate of 18% [1] and over 120,000 unsafe abortions annually [2]. Family planning (FP) is a key intervention for preventing unplanned pregnancies, unsafe abortions, and maternal death [3]. Kenya, in line with sustainable development goal 3, is implementing FP programmes aimed at universal access to sexual and reproductive health services by 2030.

Most FP interventions have been targeted towards women, who are the primary users of contraceptives. The lack of involvement of male partners results in opposition to self or partner’s FP use [4–6] due to their low level or lack of knowledge on FP. In Kenya, fear of side effects and infidelity among women using contraceptives [6, 7], misconceptions and socio-cultural concerns on FP [7] are some of the reasons for male opposition to FP use. Studies show that women often opt to use contraceptives covertly due to non-supportive spouses and relatives [4–6, 8]. However, involving both women and their partners is a strategy that promotes contraceptive acceptance, uptake and continuation [4, 9], couple communication and gender-equitable attitude [10]. Better knowledge on FP by male partners promotes cooperation; especially on the methods requiring male involvement like condoms.

Moreover, women have resorted to covert contraceptive use (CCU) due to fear of reprisal from the partner [11], religious beliefs [12] and withdrawal of support [13]; reasons that may reduce the uptake of FP for some women. Covert contraceptive use may expose women to gender-based violence if discovered [4, 5, 14] and could result in contraceptive discontinuation or change to a less preferred method [15] hence eroding the gains in increasing the contraceptive prevalence rate (CPR). A study in Senegal found that CCU is a barrier to contraceptive continuity with women deprived of appointment reminders [16] making it difficult for women to follow their contraceptive use hence increasing the risk of discontinuation. Similarly, with a rate of discontinuation at 31% [17], Kenya needs to address factors associated with and the role of CCU in discontinuation.

CCU is common in male-dominated settings where women lack the autonomy to make choices on their health [18]. Studies have shown a positive association between women empowerment and autonomy and their use of contraceptives [19–21]. Women should be free to decide and choose their preferred FP method even in settings with better male involvement and spousal communication on FP [22, 23]. However, covert use of contraceptives could highlight the discordance between women’s ability to decide on their sexual reproductive health and societal infringement of their right to choose. Previous studies in sub-Saharan Africa have shown that covert contraceptive use is associated with low levels of education [24, 25], urban residence [24, 26], richest wealth quintile [4, 24, 27] and polygamous marriage [24].

Covert contraceptive use is widespread in sub-Saharan Africa, with a prevalence of 2.6–53% [24–26, 28, 29]. In Kenya, previous studies based on the 2008/09 [24] and 2014 [1] demographic health survey (DHS), and in Nyanza region [30] found that 8.7, 7.8 and 9% of married women used contraceptives without their partners’ knowledge, but did not explore the associated factors. However, these studies reported lack of partners involvement [31], male partners resistance to contraceptive use [32], differences in fertility intentions [32] and unsupportive spouses [8] to be the reason for the CCU.

While covert use of contraceptives may contribute to the overall increase in modern contraceptive prevalence rate in Kenya, which currently stands at 58.1% [33], it could also indicate the barriers Kenyan women encounter in deciding and using FP methods and may be contributing to the 18.6% unmet need for FP [33]. With the paucity of information on CCU in Kenya, it is important to provide up to date evidence on the extent of CCU and associated factors to help in understanding contraceptive use in Kenya and contribute to informing FP interventions and policies towards Kenya’s FP goal of modern CPR of 66% by 2030 [34]. Furthermore, these findings could contribute to addressing CCU by promoting partner involvement hence reducing the risk of gender-based violence resulting from non-disclosure of contraceptive use, allaying fears of side effects, infidelity, misconceptions, and socio-cultural concerns on FP and overall increasing partners’ knowledge on FP and contraceptive use.

## Methods

### Data source and study population

We utilised data from sixth [35] and seventh [36] rounds of Kenya’s performance monitoring for accountability (PMA) surveys. The surveys used a multi-stage stratified cluster design that involved urban-rural and 11 counties (Nairobi, Bungoma, Kericho, Kiambu, Kilifi, Kitui, Nandi, Nyamira, Siaya, Kakamega and West Pokot) as strata, 151 enumeration areas (EA) sampled from the KNBS master sampling frame, 42 randomly selected households in each EA. All consenting females 15–49 years in the selected household were interviewed. Round 6 included 6106 households and 5876 females (99% response rate) while round 7 had 6097 households and 5671 females (99.1% response rate). Data were collected by trained interviewers using standardised questionnaires in November and December of 2017 and 2018 [35, 36].

### Measures

CCU, the outcome variable, was defined as “*the use of contraceptives without a male partner’s knowledge*” [37]. It was measured based on the question: “*Does your partner/husband know that you are using family planning?*” among women currently using FP and in-a-union, for which they responded either ‘*yes*’ or ‘*no*’.

The independent variables were selected based on a review of the literature on FP and the availability of the variables in the dataset. They included the county of residence, locality of residence, age in years, education levels, wealth quintiles, parity, desire for more children and age at sexual debut in years. The county of residence included the 11 counties sampled in the survey (*Nairobi, Bungoma, Kericho, Kiambu, Kilifi, Kitui, Nandi, Nyamira, Siaya, Kakamega and West Pokot*) while the locality of residence was either rural or urban, based on the classification by the KNBS master sampling framework [35, 36]. Respondents were asked how old they were on their last birthday and responses were categorized into 15–19, 20–34 and 35–49 years [27] and their highest level of education (*no formal, primary, secondary and tertiary*) [25, 27, 38]. Five wealth quintiles (*poorest, poorer, middle, richer, richest*) were computed based on wealth index generated using principal component analysis of the household assets, walls, flooring and roofing materials and type of water access and sanitation facilities [39]. Parity was assessed based on the question “*How many times have you given birth?*” and the response recoded as 0, 1, 2–3 and 4+ [25]. Women’s age at sexual debut was assessed based on the question “*How old were you when you first had sexual intercourse?*” and the response recorded in years [40]. Women were also asked whether they wanted more children, to which they responded with either ‘*yes*’, ‘*no*’ or ‘*infertile’* [29].

### Statistical analysis

We described the sample characteristics and the prevalence of CCU using frequencies and percentages. Factors associated with CCU were assessed using bivariate and multivariable logistic regressions. All variables in the bivariate analysis were included in the multivariable analysis. Stata 13.0 was used for analyses [41], which were adjusted for the sampling design and stratification using survey weight provided in the datasets. Statistical significance was set at *p-value* = 0.05.

## Results

### Sample characteristics

**Fig. 1:**
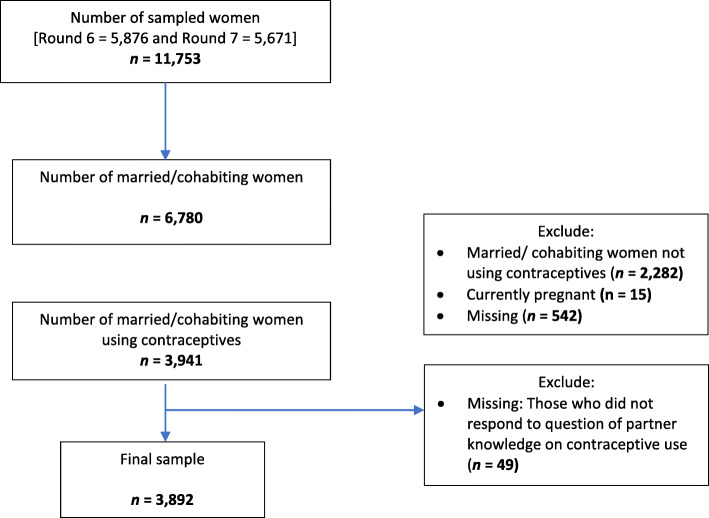
Flowchart of the study sample

Of the 11,753 sampled women, 3943 were in a union and currently using contraceptives. We included 3892 women after excluding 49 women (1.3%) who did not respond to the question on their partner’s knowledge of their contraceptive use (Fig. 1). Most participants were 20–34 years (62.9%), lived in rural areas (65.3%) mainly Bungoma (11.6%) and Kitui (11.4%) counties, and had primary level of education (51.2%) and two or more children (82.3%). The mean age at first sexual encounter was 17.3 years (standard deviation: 3.0) (Table 1). Injectables (43.7%) and implants (35%) were the commonest method of contraceptives used.

**Table 1:** Sample characteristics, and prevalence and factors associated with covert contraceptive use in Kenya (*N* = 3892)

Characteristics	Sample	Prevalence of covert contraceptive use		Bivariate logistic regression analysis		Multivariable logistic regression analysis	
	n (%)	n	% [95% CI]	COR [95% CI]	***p***-value	AOR [95% CI]	***p***-value
**County**							
Nairobi	394 (10.1)	36	9.9 [7.5–14.1]	1 (Ref)		1 (Ref)	
Bungoma	452 (11.6)	56	12.8 [9.0–17.9]	1.27 [0.75–2.17]	0.376	0.44 [0.21–0.93]	**0.031**
Kericho	406 (10.4)	76	16.7 [9.6–27.7]	1.75 [0.84–3.64]	0.135	0.68 [0.27–1.70]	0.404
Kiambu	313 (8.0)	29	9.5 [5.6–15.4]	0.90 [0.46–1.74]	0.749	0.96 [0.47–1.94]	0.900
Kilifi	268 (6.9)	27	10.1 [6.3–15.8]	0.97 [0.52–1.82]	0.931	0.31 [0.13–0.69]	**0.005**
Kitui	441 (11.3)	48	11.2 [71–18.4]	1.14 [0.59–2.17]	0.698	0.41 [0.20–0.90]	**0.025**
Nandi	386 (9.9)	28	7.2 [4.3–11.1]	0.65 [0.35–1.21]	0.177	0.23 [0.10–0.53]	**0.001**
Nyamira	410 (10.5)	44	9.2 [6.9–15.0]	0.99 [0.57–1.74]	0.979	0.37 [0.17–0.81]	**0.013**
Siaya	288 (7.4)	62	22.9 [16.7–28.1]	2.43 [1.49–3.95]	**< 0.001**	0.72 [0.36–1.46]	0.363
Kakamega	415 (10.7)	45	13.1 [7.8–19.6]	1.25 [0.66–2.36]	0.496	0.42 [0.20–0.88]	**0.021**
West Pokot	119 (3.1)	14	9.7 [4.2–18.6]	0.87 [0.35–2.12]	0.752	0.30 [0.11–0.84]	**0.022**
**Residence**							
Urban	1348 (34.6)	110	8.5 [6.7–10.8]	1 (Ref)		1 (Ref)	
Rural	2544 (65.4)	355	13.7 [11.4–16.3]	1.70 [1.22–2.36]	**0.002**	1.60 [0.99–2.58]	0.055
**Age, years**							
15–19	60 (1.5)	4	10.4 [3.0–30.2]	0.79 [0.22–2.91]	0.691	0.46 [0.10–2.18]	0.329
20–34	2446 (62.9)	282	11.9 [9.9–14.1]	0.92 [0.73–1.16]	0.528	0.92 [0.66–1.30]	0.647
35–49	1386 (35.6)	179	12.8 [10.7–15.2]	1 (Ref)		1 (Ref)	
**Education**							
No formal	90 (2.3)	20	22.8 [14.9–33.3]	5.46 [2.67–11.2]	**< 0.001**	3.79 [1.73–8.31]	**0.001**
Primary	1997 (51.3)	302	15.0 [12.3–18.3]	3.28 [1.97–5.46]	**< 0.001**	1.86 [1.06–3.29]	**0.032**
Secondary	1234 (31.7)	116	9.8 [7.8–12.2]	2.01 [1.31–3.09]	**0.002**	1.50 [0.93–2.40]	0.093
Tertiary	571 (14.7)	27	5.1 [3.4–7.7]	1 (Ref)		1 (Ref)	
**Wealth index**							
Poorest	813 (20.9)	105	18.1 [14.5–22.2]	3.18 [2.16–4.68]	**< 0.001**	2.67 [1.61–4.45]	**< 0.001**
Poorer	809 (20.8)	119	13.3 [10.7–16.4]	2.21 [1.47–3.34]	**< 0.001**	1.79 [1.05–3.04]	**0.032**
Middle	820 (21.1)	120	15.1 [11.7–19.3]	2.57 [1.71–3.86]	**< 0.001**	2.40 [1.52–3.78]	**< 0.001**
Richer	869 (22.3)	72	8.5 [6.5–11.2]	1.35 [0.89–2.05]	0.162	1.19 [0.75–1.89]	0.453
Richest	579 (14.9)	48	6.5 [4.7–8.8]	1 (Ref)		1 (Ref)	
**Parity**							
0–1	640 (17.6)	79	13.1 [10.3–16.4]	1 (Ref)		1 (Ref)	
2–3	1798 (46.2)	162	9.0 [7.1–11.3]	0.66 [0.47–0.91]	**0.013**	0.49 [0.33–0.72]	**< 0.001**
4+	1409 (36.2)	224	15.6 [13.0–18.6]	1.23 [0.91–1.65]	0.167	0.62 [0.40–0.96]	**0.033**
**Desire for more children**							
No	1815 (50.0)	240	13.3 [11.0–16.1]	1 (Ref)		1 (Ref)	
Yes	1755 (48.3)	189	11.2 [9.2–13.6]	0.82 [0.64–1.05]	0.112	0.88 [0.63–1.22]	0.434
Infertile	62 (1.7)	8	11.3 [5.9–20.7]	0.83 [0.39–1.78]	0.632	0.71 [0.31–1.63]	0.420
**Age at sexual debut (mean, SD)**	17.3 (3.0)		16.6 (2.9)	0.90 [0.86–0.95]	**< 0.001**	0.94 [0.89–0.99]	**0.029**

*COR* Crude odds ratio, *AOR* Adjusted odds ratio, *SD* Standard deviation, *Ref* Reference category: Bold: Significant at *p* = 0.05

### Prevalence of covert contraceptive use

**Fig. 2:**
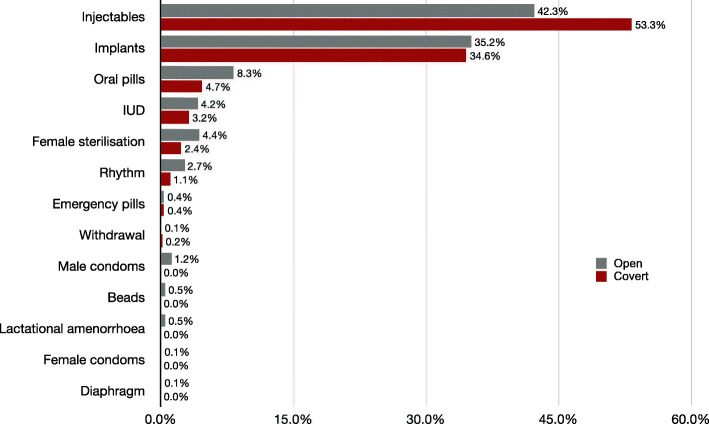
Method of contraceptive used by women with or without partners’ knowledge

The weighted prevalence of CCU was 12.2% (465/3892, 95% CI: 10.4–14.2%). It was highest among uneducated (22.8%), poorest (18.1%) and older (35–49 years, 12.8%) women; and among those living in rural areas (13.7%) and neither had children (20.8%) nor wanted for more children (13.3%). Among the counties, Siaya (21.9%), Kericho (16.8%) and Kakamega (12.6%) counties had the highest CCU prevalence (Table 1). Injectables (53.3%) and implants (34.6%) were the commonest methods of contraceptives used among covert contraceptive users (Fig. 2).

### Factors associated with covert contraceptive use

Siaya county, rural residence, education, wealth, and age at sexual debut were associated with CCU in the bivariate analysis. In the multivariable logistic regression, counties, rural residence, education, wealth, parity, and age at sexual debut were associated with CCU (Table 1). Uneducated women and those with primary education had 3.8- and 1.9-fold higher odds of CCU respectively, compared to those with tertiary education. Women from the poorest, poorer, and middle household wealth quintiles had 2.7-, 1.8- and 2.4-times higher odds of CCU compared to those from the richest quintile. Compared to women in Nairobi County, those in Kitui, Bungoma, Nyamira, West Pokot, Kilifi and Nandi counties had between 58 and 77% lower odds of CCU. Women with two or more children and those with four or more children had 51 and 38% lower odds of CCU compared to those with no children. The odds of CCU were lower by a factor of 0.94 for every year increase in age at sexual debut (Table 1).

## Discussion

The present study found a 12.2% prevalence of CCU among married women in Kenya. The prevalence was high among older, uneducated, poorest, and rural women, and among women who neither had children nor wanted for more children. The commonest methods of contraceptives used covertly were injectables and implants. Education levels, wealth, county of residence, rural residence, parity, and age at sexual debut were associated with CCU.

The prevalence of CCU in SSA ranges from 2.6 to 20.2% [24]. In our study, about one in ten women used contraceptives covertly, which signified an increase in the prevalence of CCU from 8.7%% [24] and 7.8% [1] reported in DHS of 2008/09 and 2014 respectively. The CCU prevalence is also higher than the 9% reported in a previous study in the Nyanza region of Kenya [30]. The high and increased prevalence of CCU observed in this study depicts a gap in partner involvement in contraceptive use and difficulty in communication on contraception [31, 32, 37], which are some of the main drivers of CCU in Kenya [37]. Moreover, our prevalence was higher than in Ethiopia (8.7%) [28] but lower than in Uganda (22.1%) [25]. The practice of CCU could be due to societal perception of women using contraceptive as being promiscuous [9, 12, 29, 32], patriarchy [5, 32], fear of intimate partner violence [11, 42, 43], disagreement on the number of children [32], religious beliefs [12] and limited male involvement [9, 44]. CCU could reflect women empowerment and autonomy in decision making [43] but also indicate male dominance in decision making and create a barrier to increasing contraceptive coverage [5]. The increased prevalence of CCU highlights the need for targeted interventions to improve spousal involvement in their partners’ reproductive health choices especially among the uneducated, poorest, and rural women, which will ultimately increase FP uptake and address the unmet need for FP.

Similar to previous studies in Ethiopia [28], Ghana [29], Kenya [1, 24] and Nigeria [4], injectables and implants were the commonest methods of contraceptives used overtly and covertly by married women. Injectables and implants are easy-to-use and effective [43] modern reversible contraceptives lasting 3 months, and three or 5 years, respectively [3]. Injectables are widely available and accessible in most health facilities and pharmacies, and are concealable [32] and do not require male cooperation hence promote CCU [43]. This finding highlights the need for continued support for partner involvement in FP use to promote open FP use and to ensure married women can use contraceptives that are suitable for them and not just the concealable ones. Also, a consistent availability of FP commodities in all healthcare facilities and training of health professionals to provide support to clients could help improve FP use in general.

Girls’ education delays early marriages, reduces the age at sexual debut and improves girls’ and women’s self-esteem [45], which is likely to improve communication with their partners. This could explain the observed lower odds of CCU with an increase in age at sexual debut. Women who delay their sexual debut could have a reduced need for contraceptives but also a high likelihood to use contraceptives with their partner’s knowledge. Married women with children are more likely to be involved in decision making about the desired number of children and birth spacing [25, 46] hence the lower odds of CCU among women with two or more children compared to those with none. This is consistent with a study in Ghana where women with no children were more likely to use contraceptives covertly compared to those with children [29].

We also found that women in rural areas were more likely to practice CCU compared to those in urban areas, though not statistically significant. Married urban women may be more involved in decision making on children and FP due to their high level of knowledge on contraceptive and gender-equitable attitude [23]. On the contrary, our study found that women from the rural counties of Kitui, Nyamira, West Pokot, Kilifi, Bungoma and Nandi were less likely to use contraceptives covertly compared to women in urban cosmopolitan Nairobi County. The reason for this is not clear but it may be due to perceived male dominance, reduced women autonomy cultural practices that may hinder FP uptake in general.

Our study is one of the first to explore the practice of CCU in Kenya. We used the most recent nationally representative cross-sectional data from 11 out of the 47 counties of Kenya, which makes our findings generalizable to the country. The major strength of this study is that it focuses on women-in-a-union and excludes women not-in-a-union who we could not ascertain whether they had partners. Our study findings are, therefore, important for informing immediate FP policies and interventions that focus on married couples. However, based on the cross-sectional nature of the data we could not infer causation. Also, some key determinants of CCU such as duration of the marriage, years of schooling, decision making and exposure to FP messages in the media were not collected. Regardless, our findings are comparable to those of similar settings and provide the most recent preliminary evidence on the extent and factors associated with CCU in Kenya.

## Conclusion

Our study shows that about one in 10 married women in Kenya use contraceptive covertly, with injectables and implants being the preferred methods. County of residence, education levels and rural residence were positively associated with CCU while wealth parity and age at sexual debut were negatively associated with CCU. CCU could reflect both increased or decreased women autonomy and independence in decision making on their health. While it is important to promote open contraceptive use among married couples, women should be supported to use contraceptives in whichever way they prefer. Importantly, FP programmes should promote couple’s communication and foster male involvement in FP to improve overall contraceptive use. Further research is needed to explore the reasons for CCU in Kenya, the role of unmarried women in CCU studies and whether unmarried women have the moral obligation to communicate their use of FP to their sexual partners.

## Data Availability

Data used in this study can be accessed from the Performance Monitoring for Accountability (PMA2020) website at https://www.pmadata.org/data/request-access-datasets

## Abbreviations

aOR: Adjusted odds ratio
CCU: Covert contraceptive use
cOR: Crude odds ratio
FP: Family planning
PMA: Performance Monitoring for Accountability
mCPR: Modern contraceptive prevalence rate
SD: Standard deviation
SSA: Sub-Saharan Africa

## References

[1] Kenya National Bureau of Statistics, Ministry of Health, National Council for Population and Development, ICF Macro International: Kenya Demographic and Health Survey 2014. Nairobi: Kenya National Bureau of Statistics, Ministry of Health, National Council for Population and Development and ICF Macro International; 2014:1–603. https://dhsprogram.com/pubs/pdf/fr308/fr308.pdf. Accessed 8 June 2021.

[2] Ziraba AK, Izugbara C, Levandowski BA, Gebreselassie H, Mutua M, Mohamed SF, et al. Unsafe abortion in Kenya: a cross-sectional study of abortion complication severity and associated factors. BMC Pregnancy Childbirth. 2015;15(1):34. 10.1186/s12884-015-0459-6. 10.1186/s12884-015-0459-6 PMC4338617 25884662

[3] World Health Organization (2018). Family planning: a global handbook for providers.

[4] Balogun O, Adeniran A, Fawole A, Adesina K, Aboyeji A, Adeniran P (2016). Effect of male Partner's support on spousal modern contraception in a low resource setting. Ethiop J Health Sci.

[5] Kriel Y, Milford C, Cordero J, Suleman F, Beksinska M, Steyn P, et al. Male partner influence on family planning and contraceptive use: perspectives from community members and healthcare providers in KwaZulu-Natal, South Africa. Reprod Health. 2019;16(1):89–15. 10.1186/s12978-019-0749-y. 10.1186/s12978-019-0749-y PMC6593556 31238960

[6] Ochako R, Mbondo M, Aloo S, Kaimenyi S, Thompson R, Temmerman M, et al. Barriers to modern contraceptive methods uptake among young women in Kenya: a qualitative study. BMC Public Health. 2015;15(1):118. 10.1186/s12889-015-1483-1. 10.1186/s12889-015-1483-1 PMC4336491 25884675

[7] Mwaisaka J, Gonsalves L, Thiongo M, Waithaka M, Sidha H, Agwanda A, et al. Exploring contraception myths and misconceptions among young men and women in Kwale County, Kenya. BMC Public Health. 2020;20(1):1694. 10.1186/s12889-020-09849-1. 10.1186/s12889-020-09849-1 PMC7661170 33176738

[8] Ontiri S, Mutea L, Naanyu V, Kabue M, Biesma R, Stekelenburg J (2021). A qualitative exploration of contraceptive use and discontinuation among women with an unmet need for modern contraception in Kenya. Reprod Health.

[9] Kabagenyi A, Jennings L, Reid A, Nalwadda G, Ntozi J, Atuyambe L (2014). Barriers to male involvement in contraceptive uptake and reproductive health services: a qualitative study of men and women's perceptions in two rural districts in Uganda. Reprod Health.

[10] Hartmann M, Gilles K, Shattuck D, Kerner B, Guest G (2012). Changes in Couples' communication as a result of a male-involvement family planning intervention. J Health Commun.

[11] Silverman JG, Challa S, Boyce SC, Averbach S, Raj A (2020). Associations of reproductive coercion and intimate partner violence with overt and covert family planning use among married adolescent girls in Niger. EClinicalMedicine.

[12] Mosha IH, Ruben R, Kakoko DC (2013). Family planning decisions, perceptions and gender dynamics among couples in Mwanza, Tanzania: a qualitative study. BMC Public Health.

[13] Alio AP, Daley EM, Nana PN, Duan J, Salihu HM (2009). Intimate partner violence and contraception use among women in sub-Saharan Africa. Int J Gynecol Obstet.

[14] Williams CM, Larsen U, McCloskey LA (2008). Intimate partner violence and women's contraceptive use. Violence Against Women.

[15] Cox CM, Hindin MJ, Otupiri E, Larsen-Reindorf R (2013). Understanding couples' relationship quality and contraceptive use in Kumasi, Ghana. Int Perspect Sex Reprod Health.

[16] Cavallaro FL, Duclos D, Cresswell JA, Faye S, Macleod D, Faye A, et al. Understanding 'missed appointments' for pills and injectables: a mixed methods study in Senegal. BMJ Glob Health. 2018;3(6):e000975. 10.1136/bmjgh-2018-000975. 10.1136/bmjgh-2018-000975 PMC6326323 30687521

[17] Izugbara CO, Wekesah FM, Tilahun T, Amo-Adjei J, Tsala Dimbuene ZT. Family Planning in East Africa: Trends and Dynamics. Nairobi, Kenya: African Population and Health Research Center (APHRC); 2018. https://aphrc.org/wp-content/uploads/2019/07/Family-Planning-in-East-Africa-Report_January-2018.pdf. Accessed 8 June 2021.

[18] Geleta D (2018). Femininity, masculinity and family planning decision-making among married men and women in rural Ethiopia: a qualitative study. J Afr Stud Dev.

[19] Blewussi TK, Weidert K, Kokou K, Erakalaza OB, Mensah A, Emina J, et al. Engaging men in family planning: perspectives from married men in Lomé, Togo. In: 2017 International Population Conference: IUSSP; 2017. p. 2017.

[20] Do M, Kurimoto N (2012). Women's empowerment and choice of contraceptive methods in selected African countries. Int Perspect Sex Reprod Health.

[21] Sano Y, Antabe R, Atuoye KN, Braimah JA, Galaa SZ, Luginaah I (2018). Married women’s autonomy and post-delivery modern contraceptive use in the Democratic Republic of Congo. BMC Womens Health.

[22] Beckman LJ, Harvey SM, Thorburn S, Maher JE, Burns KL (2006). Women's acceptance of the diaphragm: the role of relationship factors. J Sex Res.

[23] Bogale B, Wondafrash M, Tilahun T, Girma E (2011). Married women's decision making power on modern contraceptive use in urban and rural southern Ethiopia. BMC Public Health.

[24] Choiriyyah I, Becker S (2017). Women’s covert use of contraception in 32 countries. 2017 International Population Conference.

[25] Heck CJ, Grilo SA, Song X, Lutalo T, Nakyanjo N, Santelli JS (2018). “it is my business”: a mixed-methods analysis of covert contraceptive use among women in Rakai, Uganda. Contraception.

[26] OlaOlorun FM, Anglewicz P, Moreau C (2020). From non-use to covert and overt use of contraception: identifying community and individual factors informing Nigerian women's degree of contraceptive empowerment. PLoS One.

[27] Gasca NC, Becker S (2018). Using couple’s discordant reports to estimate female covert use of modern contraception in sub-Saharan Africa. J Biosoc Sci.

[28] Tessema BT, Hailemariam A, Reniers G: The prevalence of covert use of contraceptives in Adama town. Contraception. 2008;78(2):185. 10.1016/j.contraception.2008.04.075.

[29] Baiden F, Mensah GP, Akoto NO, Delvaux T, Appiah PC (2016). Covert contraceptive use among women attending a reproductive health clinic in a municipality in Ghana. BMC Womens Health.

[30] Harris L, Rocca C, Upadhyay U, Dworkin S, Ndunyu L, Gitome S, et al. Reproductive autonomy and covert contraceptive use in Nyanza, Kenya. Contraception. 2018;98(4):357–8. 10.1016/j.contraception.2018.07.088.

[31] Withers M, Dworkin SL, Onono M, Oyier B, Cohen CR, Bukusi EA, et al. Men's perspectives on their role in family planning in Nyanza Province, Kenya. Stud Fam Plann. 2015;46(2):201–15. 10.1111/j.1728-4465.2015.00024.x. 10.1111/j.1728-4465.2015.00024.x 26059990

[32] Harrington EK, Dworkin S, Withers M, Onono M, Kwena Z, Newmann SJ (2016). Gendered power dynamics and women's negotiation of family planning in a high HIV prevalence setting: a qualitative study of couples in western Kenya. Culture, Health Sex.

[33] Projected Trends in mCPR. https://www.familyplanning2020.org/kenya#. Accessed 8 June 2021.

[34] Family planning 2020 commitment: Government of Kenya. https://www.familyplanning2020.org/sites/default/files/Kenya_FP2020_Commitment_2017_1.pdf. Accessed 8 June 2021

[35] International Centre for Reproductive Health Kenya (ICRHK). The Bill & Melinda Gates Institute for Population and Reproductive Health at The Johns Hopkins Bloomberg School of Public Health. Kenya and Baltimore, Maryland, USA: Performance Monitoring and Accountability 2020 (PMA2020) Survey Round 6, PMA2017/Kenya-R6 Snapshot of Indicators; 2017. https://www.pmadata.org/countries/kenya/kenya-indicators/pma2017kenya-round-6-indicators.

[36] International Centre for Reproductive Health Kenya (ICRHK), The Bill & Melinda Gates Institute for Population and Reproductive Health at The Johns Hopkins Bloomberg School of Public Health: Performance Monitoring and Accountability 2020 (PMA2020) Survey Round 7, PMA2018/Kenya-R7 Snapshot of Indicators. Kenya and Baltimore, Maryland, USA; 2018. https://www.pmadata.org/countries/kenya/kenya-indicators/pma2017kenya-round-7-indicators.

[37] Biddlecom AE, Fapohunda BM (1998). Covert contraceptive use: prevalence, motivations, and consequences. Stud Fam Plan.

[38] Osuafor GN, Maputle SM, Ayiga N (2018). Factors related to married or cohabiting women’s decision to use modern contraceptive methods in Mahikeng, South Africa. Afr J Prim Health Care Fam Med.

[39] Blackstone SR, Nwaozuru U, Iwelunmor J (2017). Factors influencing contraceptive use in sub-Saharan Africa: a systematic review. Int Quart Commun Health Educ.

[40] Casey SE, Gallagher MC, Kakesa J, Kalyanpur A, Muselemu J-B, Rafanoharana RV, et al. Contraceptive use among adolescent and young women in north and south Kivu, Democratic Republic of the Congo: a cross-sectional population-based survey. PLoS Med. 2020;17(3):e1003086. 10.1371/journal.pmed.1003086. 10.1371/journal.pmed.1003086 PMC7108687 32231356

[41] StataCorp (2013). Stata Statistical Software: Release 13.

[42] McCarraher DR, Martin SL, Bailey PE (2006). The influence of method-related partner violence on covert pill use and pill discontinuation among women living in La Paz, El alto and Santa Cruz, Bolivia. J Biosoc Sci.

[43] Kibira SP, Karp C, Wood SN, Desta S, Galadanci H, Makumbi FE, et al. Covert use of contraception in three sub-Saharan African countries: a qualitative exploration of motivations and challenges. BMC Public Health. 2020;20(1):1–10. 10.1186/s12889-020-08977-y. 10.1186/s12889-020-08977-y PMC7275340 32503485

[44] Msovela J, Tengia-Kessy A (2016). Implementation and acceptability of strategies instituted for engaging men in family planning services in Kibaha district, Tanzania. Reprod Health.

[45] Marphatia AA, Saville NM, Amable GS, Manandhar DS, Cortina-Borja M, Wells JC, et al. How Much Education Is Needed to Delay Women's Age at Marriage and First Pregnancy? Front Public Health. 2020;7(396). 10.3389/fpubh.2019.00396. 10.3389/fpubh.2019.00396 PMC6964653 31993411

[46] Hameed W, Azmat SK, Ali M, Sheikh MI, Abbas G, Temmerman M, et al. Women's empowerment and contraceptive use: the role of independent versus couples' decision-making, from a lower middle income country perspective. PLoS One. 2014;9(8):e104633. 10.1371/journal.pone.0104633. 10.1371/journal.pone.0104633 PMC4131908 25119727

[47] Tsui A, Anglewicz P, Akinlose T, Srivatsan V, Akilimali P, Alzouma S, et al. Performance monitoring and accountability: the agile Project’s protocol, record and experience. Gates Open Res. 2020;4(30):30. 10.12688/gatesopenres.13119.1. 10.12688/gatesopenres.13119.1 PMC7463111 32908964

